# Selection on adaptive and maladaptive gene expression plasticity during thermal adaptation to urban heat islands

**DOI:** 10.1038/s41467-021-26334-4

**Published:** 2021-10-26

**Authors:** Shane C. Campbell-Staton, Jonathan P. Velotta, Kristin M. Winchell

**Affiliations:** 1 Department of Ecology and Evolutionary Biology, Princeton University, Princeton, NJ 08540 USA; 2 Department of Ecology and Evolutionary Biology, University of California, Los Angeles, CA 90095 USA; 3 Institute for Society and Genetics, University of California, Los Angeles, CA 90095 USA; 4 Department of Biological Sciences, University of Denver, Denver, CO 80208 USA; 5 Biology Department, Washington University, St. Louis, MO USA

**Keywords:** Evolutionary ecology, Evolutionary genetics, Regulatory networks, Animal physiology

## Abstract

Phenotypic plasticity enables a single genotype to produce multiple phenotypes in response to environmental variation. Plasticity may play a critical role in the colonization of novel environments, but its role in adaptive evolution is controversial. Here we suggest that rapid parallel regulatory adaptation of *Anolis* lizards to urban heat islands is due primarily to selection for reduced and/or reversed heat-induced plasticity that is maladaptive in urban thermal conditions. We identify evidence for polygenic selection across genes of the skeletal muscle transcriptome associated with heat tolerance. Forest lizards raised in common garden conditions exhibit heat-induced changes in expression of these genes that largely correlate with decreased heat tolerance, consistent with maladaptive regulatory response to high-temperature environments. In contrast, urban lizards display reduced gene expression plasticity after heat challenge in common garden and a significant increase in gene expression change that is congruent with greater heat tolerance, a putatively adaptive state in warmer urban environments. Genes displaying maladaptive heat-induced plasticity repeatedly show greater genetic divergence between urban and forest habitats than those displaying adaptive plasticity. These results highlight the role of selection against maladaptive regulatory plasticity during rapid adaptive modification of complex systems in the wild.

## Abstract (web summary)

Anthropogenic change, such as urban heat islands, present challenges to biodiversity that can be overcome through phenotypic plasticity. Unlike their ancestral counterparts, urban lizards have fewer maladaptive gene expression responses to higher temperatures in a common garden experiment, suggesting the evolution of adaptive plasticity.

## Introduction

Understanding the mechanisms that generate novel phenotypic diversity is a central goal of evolutionary biology. A long-standing gap in this understanding centers around the interplay between environmentally induced phenotypic variation (phenotypic plasticity) and genetically based evolutionary change. The modern history of biology has seen plasticity viewed alternatively as constraining evolution by buffering genotypes against the full brunt of natural selection^1–4^, as inconsequential for evolutionary change as a source of non-heritable phenotypic variation^5–7^, and as facilitating colonization of new environments—in particular during the incipient stages of adaptation^4^. The circumstances under which plasticity assumes these various roles^8,9^ and its role in adaptive lineage divergence^10–12^ remain controversial.

**Fig. 1:**
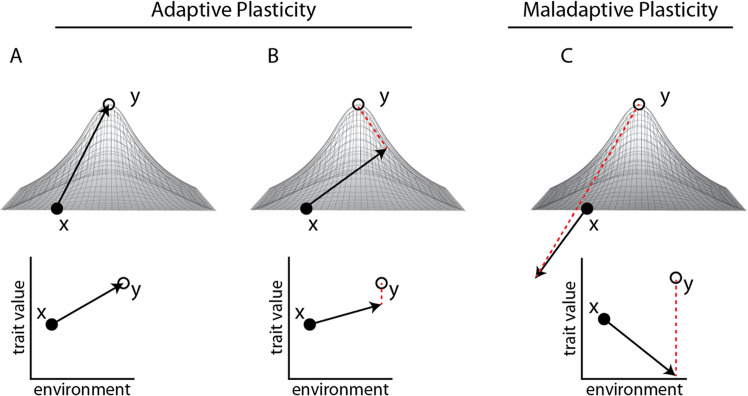
Evolution of plasticity in novel environments. Phenotypic plasticity can influence the evolutionary outcome for a population colonizing a novel environment. Ancestral plasticity may move the initial phenotype of a population (*x*) in any number of directions with respect to the local optimum of the novel environment (adaptive peak, *y*). These variable responses are adaptive when they move phenotypic values directly into (**A**) or close to—but outside—the peak *y*. If sufficient genetic variation is exposed, natural selection can reinforce adaptive plasticity and move phenotypic means towards the new peak (**B**; dotted lines). However, natural selection is not expected when plasticity puts individuals directly onto the new peak (**A**). In the case of maladaptive plasticity (**C**), inappropriate responses to the novel environment move individuals away from the peak, reducing fitness. Selection should reduce/reverse the reaction norm and restore the phenotypic mean back to the original ancestral value. In all cases, the strength of selection increases with distance from the peak (dotted lines in **B**, **C**). Based on Fig. 2 in Ghalambor et al.^4^.

The adaptive value of phenotypic plasticity depends on the heritability of environmental response and its direction of effect relative to the phenotypic optimum (Fig. 1). Adaptive plasticity moves individuals towards the phenotypic optimum in a given environment, enabling population persistence^6^ and allowing selection to act upon genetic variation underpinning the inducible response. This effect moves the population mean closer to the local adaptive peak^4,13–15^ (Fig. 1). The evolution of adaptive plasticity has been demonstrated in a wide range of developmental^16^, morphological^15^, physiological^17^, life history^13^, and behavioral phenotypes^18^.

Conversely, maladaptive plasticity results from an ancestral response that is inappropriate in a novel environment, reducing fitness^19–21^. Selection on genetic variation underlying maladaptive plasticity can minimize its magnitude and/or reverse its direction entirely^4^ (Fig. 1). For instance, ancestral physiological responses to low oxygen have evolved in lowland species to cope with acute endogenous hypoxia due to anemia, blood loss, or low tissue oxygenation^22^. However, at high altitudes, where low oxygen is exogenous and chronic, these ancestral responses result in maladaptive pathologies including pulmonary hypertension and an overproduction of red blood cells (polycythemia)^22^. As a result, many species native to high-altitude environments have evolved a reduction or complete loss (canalization) of this ancestral plasticity^23–27^.

Acclimation and evolutionary adaptation to environmental change often involves complex coordinated biological responses, such as the co-regulation of genes underpinning physiological function. Reinforcement of adaptive plasticity and reduction/reversal of maladaptive plasticity at the regulatory and genomic levels likely occur in tandem to drive populations toward new adaptive peaks^28^. However, the relative importance of adaptive and maladaptive plasticity as substrates for selection during the incipient stages of lineage divergence remains largely unexplored.

Although identifying the initial mechanisms that drive adaptive divergence is critical for understanding the evolution of complex systems in novel environments, studying such phenomena becomes more difficult as the time since lineage divergence proceeds, due to the accumulation of mutations, complex demographic histories, and subsequent selective events. Examples of contemporary evolution, such as urban adaptation, provide “natural experiments” that offer unique insights into the mechanisms that underpin the evolution of complex environmentally-responsive traits. Such examples provide means to quantitatively explore evolutionary dynamics during the initial stages of lineage divergence and local adaptation in the wild^29,30^. Further, disentangling the interactions among selection, adaptation, and plasticity has become increasingly important for understanding the evolutionary implications of human-induced environmental alteration^30^. Rapid habitat modification and environmental shifts due to direct and indirect human influence may induce evolution of physiological, behavioral, and/or developmental plasticity^31–34^. Ancestral plasticity may therefore be a common target of natural selection if it results in greater population resilience to climate change and anthropogenic habitat alteration^32,33,35^.

One such anthropogenic habitat alteration is the increase in ambient temperature in urban areas due to increased impervious surface cover and heat production, collectively termed the “urban heat island effect”^36^. For ectothermic species in particular, elevated urban temperatures have profound impacts on performance^37–42^, reproduction, development^43–45^, and survival^46–49^. A growing body of evidence supports resilience to high temperatures as a key facet of urban adaptation for terrestrial ectotherms^37–39,41,42,50^. Furthermore, ancestral plasticity of heat stress responses aid in short-term survival of individuals exposed to periodic acute temperature spikes and may enable initial population persistence in novel urban environments^51^. For instance, behavioral thermoregulation can allow individuals to achieve optimal body temperatures by exploiting thermal heterogeneity, thereby minimizing selection on thermal physiology^52^ (Bogert Effect^53^). However, in extreme environments, temperatures may fall outside the ancestral range of variability or provide inadequate thermal heterogeneity for behavioral thermoregulation. As a result, these novel extreme thermal environments may necessitate physiological adaptation. In such cases, plastic physiological response to short-term thermal variation can result in tradeoffs with other traits critical for survival and reproduction^54,55^, including diminished growth rates, body condition, clutch size, and locomotor performance^56–59^. Colonization of urban heat islands, where heat stress is likely to be common, chronic, and extreme, may exacerbate the maladaptive consequences of plastic physiological response. Consequently, populations colonizing urban environments may face the multifaceted physiological conundrum of employing heat-induced plasticity that contributes to greater physiological resilience at high temperatures, while mitigating associated maladaptive consequences.

**Fig. 2:**
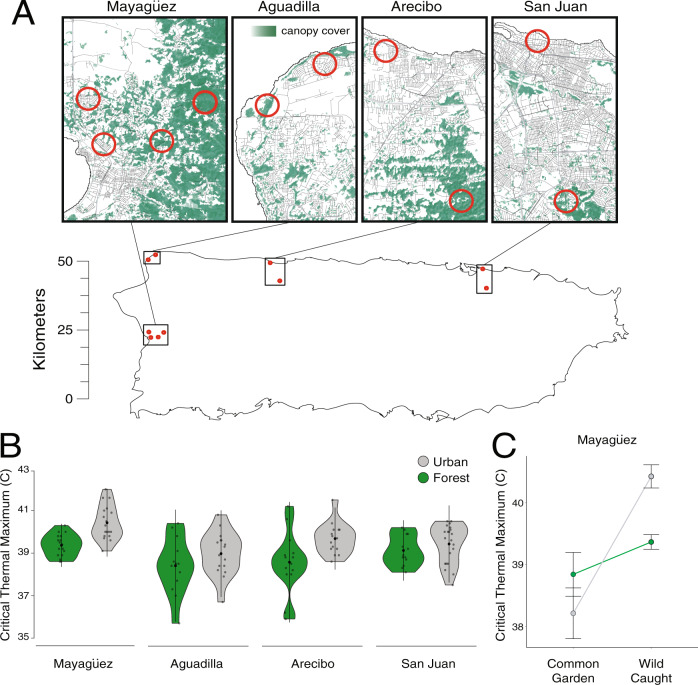
Divergent heat tolerance between urban and forest populations. **A** Map of Puerto Rico and collection sites^60^. **B** Violin plots of heat tolerance (Critical Thermal Maximum, CT_MAX_) measured for wild-caught lizards from forest (green) and urban (gray) populations in four municipalities (Aguadilla: forest *n* = 16, urban *n* = 16; Arecibo: forest *n* = 21, urban *n* = 20; Mayagüez: forest *n* = 18, urban *n* = 18; San Juan: forest *n* = 16, urban *n* = 25). Wild-caught urban populations display significantly greater heat tolerance than their forest counterparts across municipalities (linear mixed-effects model: 0.8247 ± 0.1771, *χ*^2^ = 20.093, *p* < 0.001, effect size = 0.337^60^). Black dots and vertical lines in the center of each violin represent the mean and SD, respectively. **C** Comparison of CT_MAX_ between urban (gray) and forest (green) lizards from Mayagüez born and raised in common garden (*n* = 16; forest: 25 °C *n* = 3, 32 °C = 4) (urban: 25 °C *n* = 5, 32 °C = 4) vs. wild-caught (*n* = 36, forest *n* = 18, urban *n* = 18). Dots and black bars represent mean ± 1 SE. Lines connecting treatments highlight the magnitude of mean CT_MAX_ difference between common garden and native environments. No significant differences in CT_MAX_ are observed between urban and forest lineages under common garden conditions, supporting plastic response to divergent native thermal environments as the primary source of observed in situ differences. Ancestral variation in this plastic response may be the target of selection in the novel thermal environments of urban heat islands.

The Puerto Rican crested anole (*Anolis cristatellus*) is emerging as a powerful model to understand the interaction between natural selection and plasticity during the early stages of adaptation to urban heat islands. Urban habitat modification has been ongoing across Puerto Rico since European colonization five centuries ago and *A. cristatellus* has occupied urban habitats for at least 35 generations (Supplementary Table 1). Campbell-Staton et al.^60^ finds that wild-caught lizards in cities across the island display greater heat tolerance when compared to closely related forest counterparts (Fig. 2A, B). Urban and forest lineages do not display this physiological divergence when born and raised in common garden environments (Fig. 2C), highlighting plasticity of thermal response to native environments as the primary source of observed physiological divergence in the wild.

These findings set up two distinct hypotheses regarding the role of thermal plasticity in facilitating colonization of urban heat islands: first, ancestral thermal plasticity exhibited by forest populations may be completely sufficient to obtain optimal performance at novel urban temperatures (perfect plasticity hypothesis). Alternatively, plasticity of forest populations may be insufficient to reach the new thermal optimum in urban environments and require the additional action of selection modifying genetic variation underlying thermal plasticity to move the colonizing populations closer to the adaptive peak of the novel environment (evolved plasticity hypothesis). The major distinction between these alternative hypotheses is the action of selection on the genetic underpinnings of thermal plasticity. Campbell-Staton et al.^60^ reports parallel patterns of elevated genetic divergence associated with large suites of temperature-responsive genes between urban and forest habitats, and identifies a single nonsynonymous polymorphism that displays genotype-by-environment interactions associated with elevated thermal tolerance, but only within urban heat islands^60^. The persistent and repeated signatures of genomic selection associated with thermal plasticity observed in the wild are congruent with the evolved plasticity hypothesis as a significant contributor to differences in thermal resilience observed between environments^60^. However, it remains unclear how selection acts on standing variation underlying this regulatory plasticity to facilitate rapid alterations of thermal performance.

**Fig. 3:**
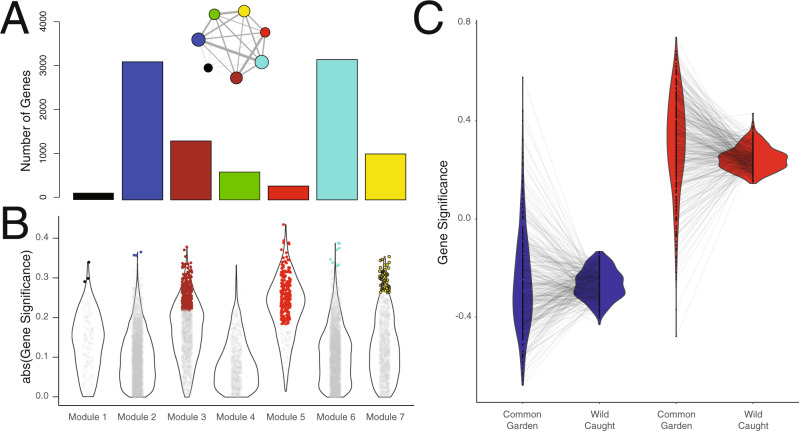
Identification of genes associated with heat tolerance in common garden and wild-caught lizards. **A** One hundred and thirty lizards from heterogeneous treatments (114 wild-caught, 16 common garden) were used to identify co-expression modules of the *Anolis* skeletal muscle transcriptome. Weighted gene correlation network analysis (WGCNA) revealed seven regulatory modules that define the full regulatory architecture of the skeletal muscle transcriptome: Module 1 (black), Module 2 (blue), Module 3 (brown), Module 4 (green), Module 5 (red), Module 6 (turquoise), and Module 8 (yellow)^60^. Bars represent the number of genes within each module. Inset is a representation of connectivity among co-expression modules. Circle sizes represent the relative size of each module (circle area is proportional to log-transformed number of genes within each module) and line thickness represents the relative strength of connectivity between modules (absolute value of pairwise Pearson’s correlation among eigengene values). **B** Violin plot of gene significance scores (GS) for CT_MAX_ (strength of association between gene expression and CT_MAX_). Dots indicate individual genes assigned to each module. Colored dots within each module indicate genes with significant phenotypic correlations after multiple testing correction (*GS.q* < 0.05). This subset of genes made up the focal data set of the current study. **C** To validate the candidate gene set, two additional network analyses were conducted for wild-caught (*n* = 114) and common garden lizards (*n* = 16), separately. Gene significance (GS) scores were calculated for candidate genes independently for each group. Genes identified as negatively correlated with CT_MAX_ (negative regulators) in the full data set are indicated in blue; genes positively correlated with CT_MAX_ in the full data set (positive regulators) are indicated in red. Black dots in the center of each violin plot are GS scores for each individual gene. Gray lines present the directionality of GS score change for each gene between common garden and wild-caught animals. There was a significant expression-phenotype correlation between wild-caught and common garden data (linear model, *R*^2^: 0.601; *p* « 0.001). Relative rank of gene significance was highly conserved across sets (Spearman’s rank correlation *ρ*: 0.75, *p* « 0.001). Negative regulators displayed no bias in gene significance between groups (paired *t*-test, *p* = 0.26). Positive regulators displayed higher gene significance values in the common garden data set than the wild-caught data set (paired *t*-test, *p* « 0.001). The gene–gene correlations between the wild-caught and common garden data sets was small but statistically significant in each case (positive regulators: *R*^2^: 0.04, *p* < 0.0001; negative regulators *R*^2^: 0.03, *p* = 0.003).

**Fig. 4:**
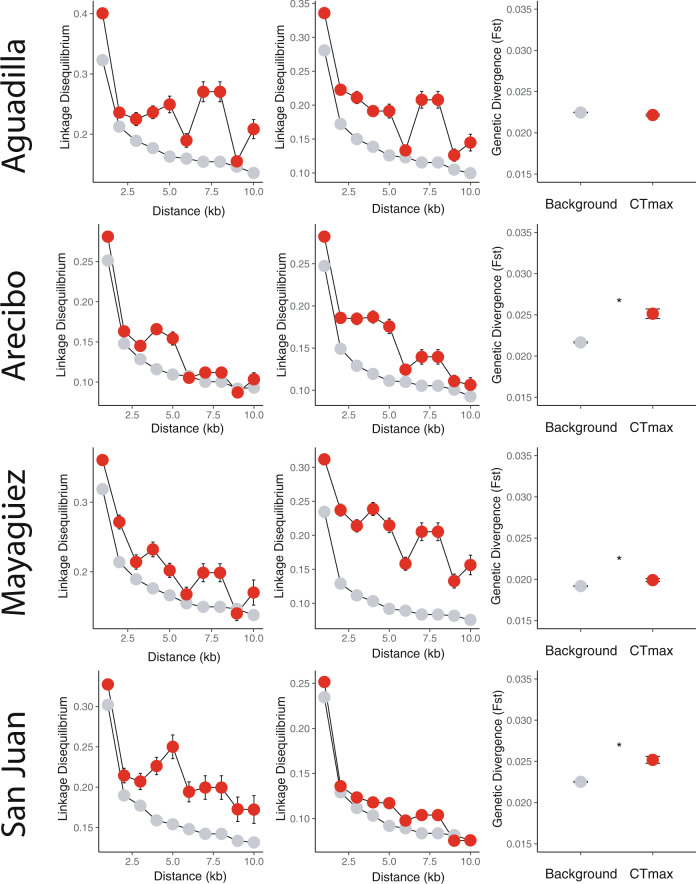
Signatures of polygenic selection on CT_MAX_-associated SNPs. Extended linkage disequilibrium (≥5 kb) is observed in CT_MAX_-associated SNPs (red) in comparison to the transcriptome-wide background (gray) (linear mixed-effects model*: p* < 0.05) in both forest (Column 1) and urban (Column 2) habitats, suggesting ongoing selection on thermal physiology island-wide. In addition, increased genetic divergence between urban and forest habitats (Column 3) in CT_MAX_-associated SNPs (red) with respect to background (gray) expectations (Arecibo, Mayagüez, and San Juan: Welch *t*-test; *p* < 0.05 (asterisks)) supports selection on heat tolerance specific to urban heat islands. LD plots are represented in 1 kb distance bins. Dots and error bars represent means ± 1 SE. All 130 animals for which we had sequence data were used for this analysis.

In this study, we build upon the findings of Campbell-Staton et al.^60^ to examine the relative roles of adaptive and maladaptive gene expression plasticity as targets of selection in urban habitats. First, using the gene expression data from Campbell-Staton et al.^60^, we characterize the regulatory underpinnings of temperature-dependent performance in *A. cristatellus* to identify a set of candidate genes within the skeletal muscle transcriptome of the hind limb displaying significant correlations with heat tolerance (CT_MAX_, Fig. 3A, B and Supplementary Fig. S2); this candidate set serves as a proxy for transcriptional drivers of thermal performance, representing the combined contributions of phenotypic plasticity (developmental plasticity and phenotypic flexibility) and evolved divergence (Fig. 4). Next, we explore evolved divergence in plasticity between urban and forest lineages by assessing the relative contributions of adaptive and maladaptive plasticity to candidate gene expression, using lizards born and raised under common laboratory conditions. Finally, we compare genetic divergence and mechanisms of selection associated with adaptive and maladaptive gene expression plasticity in four pairs of urban–forest populations. We do this by measuring the relative magnitude of genetic divergence between habitat types for genes associated with each plasticity category (adaptive or maladaptive, Fig. 7) and identify putative molecular targets of selection in each case (Fig. 8). Using these sequential and complementary analyses, we suggest that rapid adaptation of *Anolis* lizards to urban heat islands is facilitated primarily by selection for reduced and/or reversed heat-induced plasticity that is maladaptive in urban thermal environments.

## Results and discussion

### Identifying regulatory underpinnings of temperature-dependent performance

We present novel and extended analyses of the data collected by Campbell-Staton et al.^60^, to further investigate transcriptome-wide regulatory evolution in hind limb skeletal muscle, an essential component of locomotion^61,62^, displaying repeated patterns of temperature-dependent plasticity and signatures of selection in urban heat islands. In brief, Campbell-Staton et al.^60^ measured CT_MAX_ for a panel of wild-caught lizards from four urban–forest pairs of *A. cristatellus* from the municipalities of Aguadilla, Arecibo, Mayagüez, and San Juan, which represent independent urban colonization events across the island of Puerto Rico (*n* = 114^60^). Critical thermal maximum (CT_MAX_) is a measure of physiological response to acute temperature change^63^ that estimates the temperature at which an organism experiences systemic dysfunction—an inability to coordinate locomotor performance^64^. In addition, CT_MAX_ was measured for urban and forest lizards from Mayagüez born and raised in common laboratory conditions (*n* = 16). Subsequent to CT_MAX_ trials, wild-caught individuals were exposed to one of three 2 h temperature treatments at average field-measured body temperatures from forest (day treatment: 25.4 ± 0.614 °C; night treatment: 15.49 ± 1.82 °C) or urban (day treatment: 32.09 ± 3.1 °C) habitats. Common garden individuals were randomly split between the forest day and urban day treatments only. After the acclimation period, skeletal muscle transcriptomes were collected for RNA sequencing (RNAseq) analyses (see “Methods,” and Supplementary Fig. 2).

To categorize the regulatory architecture of the *Anolis* skeletal muscle transcriptome, we then analyzed the resultant expression data for all individuals (wild-caught *n* = 114, common garden = 16, Supplementary Fig. 1) using weighted gene correlation network analysis (WGCNA^65^). Weighted correlation network analysis is a systems biology method for identifying groups of highly co-expressed genes (modules), summarizing module-level expression, identifying intramodular genes of importance (hub genes), and relating expression patterns to trait variation^65^. With this approach, we identified genes across the skeletal muscle transcriptome (total gene *n* = 11,654) whose expression was significantly associated with CT_MAX_ in *A. cristatellus* using the gene significance function (GS^65^). To account for modular structure within the transcriptome, we separated genes into seven gene expression modules, which define the entire regulatory architecture identified by WGCNA^60^ (Fig. 3A, B). Across these modules, we identified a total of 632 genes with expression values significantly associated with CT_MAX_, after correcting for multiple testing (*GS.q* < 0.05) (Fig. 3B). This set of CT_MAX_-associated genes are considered putative candidate genes for thermal performance or all subsequent analyses.

Functional enrichment analysis of these candidate genes revealed enrichment of biological processes associated with cellular heat shock response. Three interrelated biological processes (GO:0044089—positive regulation of cellular component biogenesis, *p* = 0.036; GO:0045898—regulation of RNA polymerase II transcriptional preinitiation complex assembly, *p* = 0.0004; GO:0044265—cellular macromolecule catabolic process, *p* = 0.004) share a core set of genes associated with the RNAII transcription preinitiation complex (Supplementary Table 2), which plays a critical role in regulating cellular response to heat shock by initiating transcription of genes that produce heat shock proteins^66,67^. A fourth biological process (GO:0007059—chromosome segregation, *p* = 0.025) contains a suite of genes involved in DNA replication and repair, which are critical for maintenance of DNA integrity during and after cellular heat shock^68^. These genes include several that encode kinesin, condensin, kinetochore, RNA helicase, and type II DNA topoisomerase proteins (Supplementary Table 2). Together, these functional associations highlight potential cellular mechanisms underpinning individual variation in heat tolerance and potential targets of selection within urban heat islands.

We combined all individuals from common garden and wild-caught groups for initial candidate gene identification, in order to recover the most robust set of thermal tolerance-associated candidate genes in the species. Consequently, our candidate gene set captures expression-phenotype correlations due to the combined effects of developmental thermal environment (developmental plasticity), temperature-dependent expression in adult individuals (phenotypic flexibility), and interactions therein. Capturing this variation in potential regulatory contributions was deemed critical for subsequent analyses, as selection on any of these components may play an important role in adaptive temperature-dependent gene expression divergence between lineages.

We next validated the ability of these candidate genes to recapitulate expression-phenotype correlations independently in wild and common garden environments. We re-ran WGCNA twice more as outlined above, separating individuals from the common garden and wild-caught groups. We then recalculated GS for each candidate gene and compared them across both data sets (Fig. 3C). There was a significant correlation between expression-phenotype values of the wild-caught and common garden data (linear model, adjusted *R*^2^: 0.601; *p* « 0.001). Relative rank of GS was also highly conserved across groups (Spearman’s rank correlation *ρ*: 0.631, *p* « 0.001). Among genes that displayed negative correlations with CT_MAX_ (negative regulators), we observed no significant differences in GS between wild and common garden conditions (paired *t*-test, *t* = 0.81, df = 295, *p* = 0.42). Genes positively correlated with CT_MAX_ (positive regulators) displayed significantly higher GS in common garden than in the wild (paired *t*-test, *t* = 5.58, df = 335, *p* « 0.001), suggesting a greater ability to predict expression-phenotype relationships in common garden. Higher variance was observed in the common garden data set, resulting in lower explanatory power within each regulatory category. However, the correlation between the wild-caught and common garden data sets remained significant in each case (positive regulators: *R*^2^: 0.04, *p* < 0.0002; negative regulators *R*^2^: 0.03, *p* = 0.0003). In summation, our candidate gene set displays similar expression-phenotype correlations and relative rank correlations between common garden and wild-caught individuals, supporting the gene set as suitable for analysis in both native and common garden environments.

To directly assess evolutionary contributions to expression variation in these candidate genes, we searched for genomic signatures of selection in each population using all individuals (wild-caught = 114, common garden = 16). If the plasticity producing observed differences in thermal tolerance in the wild are the target of selection in urban habitats, we would expect to see significant signatures of selection at the sequence level specific to our candidate loci. We identified 2,161,083 single-nucleotide polymorphisms (SNPs) transcriptome-wide using RNAseq reads from lineages originating in all four municipalities studied in Campbell-Staton et al.^60^ (Aguadilla: forest *n* = 11, urban *n* = 16; Arecibo: forest *n* = 18, urban *n* = 16; Mayagüez: forest *n* = 19, urban *n* = 18; San Juan: forest *n* = 11, urban *n* = 21). Using all polymorphic loci within candidate genes as the focal candidate SNP set, we tested for significant deviations from neutrality by direct comparison with the null expectation estimated by background variation^69^ across all SNPs within genes that had no significant association with CT_MAX_ (Fig. 4).

We calculated pairwise linkage disequilibrium (LD; *R*^2^ within non-overlapping 10 kb windows) and tested for deviations from Hardy–Weinberg (HW) equilibrium across all polymorphic sites of the transcriptome. We then compared patterns of LD (binned in 1 kb increments) between candidate and background SNP sets. As LD and HW equilibrium can be influenced by both selection and demography, we restricted our analyses to comparisons within each site such that the background and candidate SNP sets shared a common demographic history. If our candidate genes have been a common target of selection, we would expect to observe a significant increase in LD at polymorphic sites proximate to these genes when compared to the transcriptome-wide background. Consistent with this expectation, we found that SNPs within CT_MAX_-associated genes displayed significantly elevated LD (linear mixed-effects model: *p* < 0.05, Fig. 4) across all sampled localities. Significant deviations from HW equilibrium were also more prevalent among SNPs within CT_MAX_-associated genes compared to the background SNP set (equality of proportions test—Arecibo: *χ*^2^ = 77.721, df = 1, *p*-value << 0.001; Aguadilla: *χ*^2^ = 28.478, df = 1, *p*-value << 0.001; Mayagüez: *χ*^2^ = 37.71, df = 1, *p*-value << 0.001; San Juan: *χ*^2^ = 33.383, df = 1, *p*-value = << 0.001; Supplementary Table 3).

These genomic patterns suggest that the regulatory underpinnings of heat tolerance are broadly under selection across the range of *A. cristatellus*, supporting previous work demonstrating the integral role of thermal biology in habitat specialization among *Anolis* occupying divergent macro- and microclimatic niches^70–72^. If, in addition to this broader selection on thermal physiology, populations are under significant contemporary selection within urban heat islands, we would also expect to observe increased genetic divergence (*F*_ST_) across urban–forest boundaries when comparing SNPs within candidate loci to null expectations from the transcriptome-wide background. We found this to be the case in three of our four urban–forest comparisons (Arecibo, Mayagüez, and San Juan: Welch two-sample *t*-test, *p* << 0.001, Fig. 4), suggesting that CT_MAX_-associated genes are repeated targets of selection between urban and forest habitats across the island.

### Evolved divergence in gene expression plasticity between urban and forest lineages tested with a common garden experiment

**Fig. 5:**
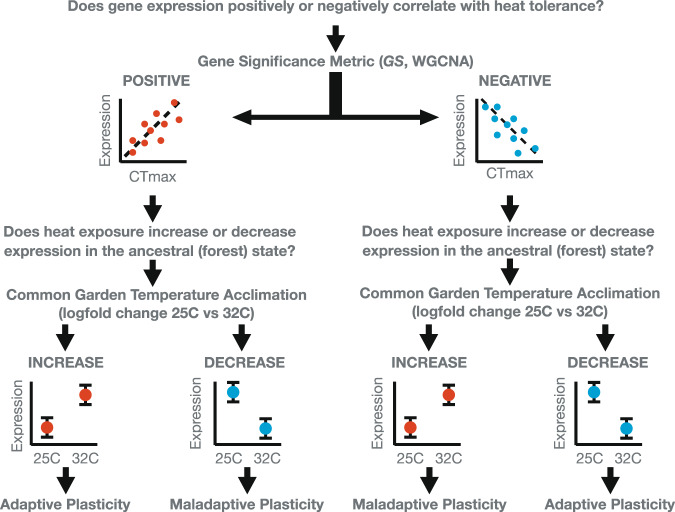
Theoretical workflow for categorizing the ancestral plastic response of a given gene to increased urban temperatures as adaptive or maladaptive. First, the direction of gene expression correlation (measured from a panel of 130 lizards) was used to identify a given gene as a positive regulator (positively correlated with heat tolerance, CT_MAX_) or a negative regulator (negatively correlated with CT_MAX_). Next, the gene was categorized as upregulated (increasing in expression) or downregulated (decreasing in expression) in forest lineages born and raised in common conditions (a proxy for the ancestral condition) in response to increased temperature (25 °C–32 °C). Theoretical dots and bars represent mean ± 1 SE. Positive regulators displaying increased expression at high temperature and negative regulators displaying decreased expression at high temperature in common garden lizards were categorized as displaying adaptive plasticity. Decreased expression of positive regulators at high temperature and increased expression of negative regulators at high temperature in common garden individuals were deemed maladaptive plasticity.

To categorize heat-induced plasticity of a given gene as adaptive or maladaptive, we made the assumption that greater heat tolerance is adaptive at higher temperatures^51^, whereas lower heat tolerance is maladaptive, resulting in reduced fitness^73,74^. Using the GS scores from the WGCNA analyses outlined above, we categorized the direction of expression-phenotype correlation as positive or negative for each CT_MAX_-associated candidate gene. We then characterized differences in expression observed for each common garden group exposed for 2 h, to either average forest (25 °C; forest *n* = 4, urban *n* = 5) or average urban (32 °C, forest *n* = 3, urban *n* = 4) body temperatures, as congruent (adaptive) or incongruent (maladaptive) with increased heat tolerance. Cases in which the direction of common garden plasticity matched the expectation for greater CT_MAX_ were deemed putatively adaptive. Those that opposed this expectation were deemed putatively maladaptive. For an outline of the categorization workflow, see Fig. 5. Forest lineages of *A. cristatellus* predate anthropogenic habitat modification. Therefore, we used the common garden forest group (*n* = 7; 25 °C *n* = 3, 32 °C = 4) as a proxy for the ancestral state of gene expression for comparison against the derived (urban) gene expression state (*n* = 9, 25 °C *n* = 5, 32 °C = 4).

**Fig. 6:**
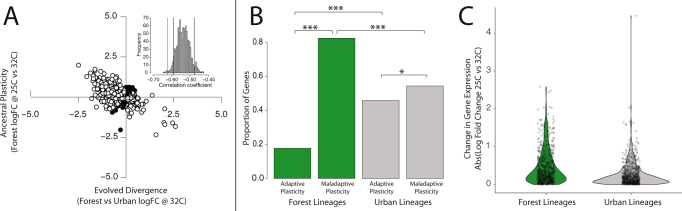
Evolved gene expression divergence between urban and forest lineages measured through a common garden experiment. Lizards from urban and forest lineages from Mayagüez were born and raised in a common garden laboratory setting to study patterns of evolved divergence in gene expression between habitats types. **A** The relationship between ancestral (forest) plasticity of gene expression (25 °C vs. 32 °C) and evolved divergence in gene expression under heat challenge (forest vs urban at 32 °C). Each dot represents a single gene from the focal set of CT_MAX_-associated genes (*n* = 632). Dots displayed in black represent genes with a positive correlation between ancestral plasticity and evolved divergence. Those displayed in white represent genes with a negative association between ancestral plasticity and evolved divergence (Spearman’s *ρ* = −0.61). Among genes associated with heat tolerance, evolved divergence reverses the direction of ancestral plasticity. Inset: the observed correlation coefficient (red line) is more negative than the lower 95th percentile of a randomized null distribution (black line). **B** Bar graph representing the number of genes displaying adaptive vs. maladaptive plasticity in common garden lineages from forest (green) and urban (gray) habitats. Figure 5 outlines the methodology of plasticity categorization. Among genes associated with heat tolerance, urban lineages possess a significantly larger proportion displaying adaptive plasticity and a significantly lower proportion that displaying a maladaptive plasticity in response to increased temperature. Asterisks represent degree of significance (equality of proportions test within habitat types, exact binomial test between habitat types: *p < 0.05, ***p < 0.001). **C** Violin plot displaying heat-induced regulatory plasticity (log-fold change in expression from 25 °C to 32 °C) in forest (green) and urban (gray) lineages. Individual genes are represented by gray dots. Urban lineages display an overall lower magnitude of gene expression plasticity (Welch two-sample *t*-test: *p* < 0.001), congruent with evolutionary attenuation of maladaptive plasticity. Sixteen animals from common garden conditions were used for these analyses (25 °C; forest *n* = 4, urban *n* = 5) or average urban (32 °C, forest *n* = 3, urban *n* = 4).

We found that urban and forest groups from Mayagüez diverged significantly in heat-induced gene expression plasticity under common garden conditions, supporting evolved divergence between lineages. Forest lineages displayed a disproportionately maladaptive response to heat stress (82.3% of genes, *n* = 520, binomial test: *p* << 0.001, Fig. 6A, B), with the majority of positive regulators (87.2%) being downregulated (*n* = 293, *p* << 0.001) and the majority of negative regulators (76.7%) being upregulated (*n* = 227, *p* << 0.001) in response to increased temperature. These results indicate ancestral heat-induced plasticity of gene expression is predominantly associated with lower CT_MAX_ and may therefore be maladaptive for physiological performance in urban heat islands. In contrast, urban lizards from Mayagüez displayed significantly less maladaptive plasticity (forest = 82.3%, urban = 54.3%, exact binomial *p* < 0.001) and significantly more adaptive plasticity (forest = 17.7%, urban = 45.7%, exact binomial *p* < 0.001) across candidate genes when exposed to the urban temperature treatment (Fig. 6B).

The observed divergence in proportions of adaptive and maladaptive responses in common garden lineages suggests that selection in urban habitats has acted primarily to reduce and/or reverse maladaptive gene expression plasticity. Under such a scenario, we would expect to observe an overall reduction of heat-induced gene expression plasticity in derived urban lineages^75^. As a result, the direction of evolutionary divergence should be negatively correlated with the direction of ancestral plasticity^75^. To test this hypothesis, we estimated ancestral plasticity of a given gene as the log-fold change of expression in common garden forest lizards across the two temperature treatments (25 °C *n* = 3, 32 °C = 4) and evolved divergence as the log-fold change in expression between the forest and urban common garden groups at 32 °C (urban *n* = 4, forest *n* = 4). We then quantified the direction and strength of the correlation between ancestral plasticity and evolved divergence across the entire CT_MAX_-associated candidate gene set for the Mayagüez populations.

Consistent with expectations of predominant selection against maladaptive plasticity, ancestral plasticity and evolved divergence displayed a strong negative correlation (Spearman’s *ρ* = −0.61; 82.3% of genes, *n* = 520, equality of proportions test, *p* < 0.001, Fig. 6A). To account for potential statistical artifact due to regression towards the mean^76^, we conducted a randomization test to estimate a null distribution of correlation coefficients and found that the strength of our observed correlation was significantly more negative than expected as a result of artifact alone (empirical *p* < 0.05, Fig. 6A inset). If selection has acted primarily to reduce the magnitude of maladaptive plasticity, we would also expect urban lineages to display a significant reduction in the magnitude of heat-induced gene expression response when compared to forest counterparts. Therefore, we directly compared the magnitude of heat-induced plasticity between common garden forest and urban groups at 25 °C vs. 32 °C (forest: 25 °C *n* = 3, 32 °C = 4; urban: 25 °C *n* = 5, 32 °C = 4) and found that urban lizards displayed a blunted heat-induced gene expression response (lower log-fold change in expression) compared to forest lizards (Welch two-sample *t*-test, *p* < 0.001, Fig. 6C). Taken together, the differences observed between forest and urban groups from Mayagüez in common garden support evolutionary divergence, driven primarily by the reduction and/or reversal of maladaptive plasticity present in ancestral forest lineages.

### Comparing genetic divergence and mechanisms of selection for adaptive and maladaptive gene expression plasticity

Results of the common garden experiment suggest that temperature-dependent selection against maladaptive plasticity has played a predominant role in evolutionary gene expression divergence between urban and forest populations of *A. cristatellus*. Under this hypothesis, we would expect genetic divergence among genes displaying maladaptive plasticity to exceed genetic divergence associated adaptive plasticity and neutral rates of divergence observed broadly across the rest of the transcriptome. Furthermore, differences in the rate of genetic divergence observed across plasticity categories may suggest different targets of selection acting to drive local adaptation in urban environments.

Therefore, we directly compared patterns of genetic divergence between urban and forest habitats to test the prediction that loci underpinning maladaptive plasticity diverge more rapidly between novel (urban) and ancestral (forest) lineages compared to those underpinning adaptive plasticity. To test this prediction, we divided CT_MAX_-associated candidate SNPs into those occurring within the putatively adaptive and maladaptive plasticity categories prescribed above. Significantly elevated genetic divergence (*F*_ST_) and enrichment of *F*_ST_ outliers observed within the candidate SNP set beyond the background expectation would provide evidence for selection acting on this group of interacting phenotype-associated genes (polygenic selection)^77,78^.

**Fig. 7:**
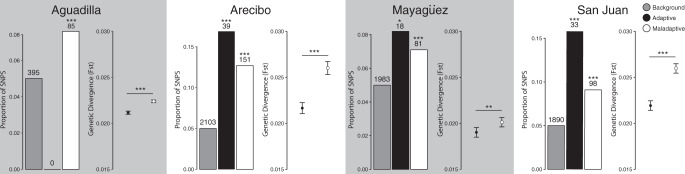
Repeated increases in urban–forest genetic divergence associated with maladaptive plasticity. Comparisons of genetic divergence (*F*_ST_) among heat tolerance-associated genes in four municipalities, representing independent urban colonization events across Puerto Rico^58^. Bar graphs display the proportion single-nucleotide polymorphisms (SNPs) within the adaptive (black) and maladaptive (white) gene sets that occur beyond the *p* = 0.05 empirical distribution of genetic divergence (*F*_ST_), based on the transcriptome-wide background (gray). Asterisks indicate a significant enrichment of outliers within a gene class (*p < 0.05, **p < 0.01, ***p < 0.001, two-sample equality of proportions test with continuity correction). No outlier gene SNPs were found in the adaptive plasticity gene set within Aguadilla. Dot plots display mean ± 1 SE of genetic divergence (*F*_ST_) for SNPs within the adaptive (black) and maladaptive (white) plasticity gene classes. Asterisks indicate significant differences in mean divergence (*p < 0.05, **p < 0.01, ***p < 0.001, Welch two-sample *t*-test). All 130 animals for which we had sequence data were used in this analysis.

We first quantified genetic divergence associated with plasticity within each pair of urban–forest populations by testing for statistical enrichment of *F*_ST_ outliers within the adaptive and maladaptive plasticity classes. These comparisons of genetic divergence were conducted independently for each of the four paired urban–forest groups using sequence data from all individuals (wild-caught *n* = 114, common garden *n* = 16) representing each municipality. We used a *p* < 0.05 threshold based on the empirical distribution of genetic divergence for the transcriptome-wide background. We found that genes displaying adaptive plasticity were significantly enriched for *F*_ST_ outliers in three of the four urban–forest comparisons (Arecibo, Mayagüez, and San Juan), whereas genes displaying maladaptive plasticity were significantly enriched for outliers in all four comparisons (Fig. 7). These results support the action of selection on both adaptive and maladaptive gene expression plasticity.

We then assessed the relative strength of selection on adaptive and maladaptive plasticity by comparing mean *F*_ST_ between each urban–forest pair (wild-caught *n* = 114, common garden *n* = 16). We found that genes associated with maladaptive plasticity had greater genetic divergence across urban–forest boundaries than those displaying adaptive plasticity in all four municipalities (Welch two-sample *t*-test, *p* < 0.01) (Fig. 7). Although both adaptive and maladaptive plasticity appear to be common targets of selection in urban heat islands, these data suggest that selection operates more intensely on maladaptive plasticity during the initial stages of genetic divergence across urban–forest boundaries.

Finally, we examined potential genetic mechanisms underpinning adaptive and maladaptive plasticity by evaluating the proportion of noncoding and coding polymorphisms associated with genetic divergence between forest and urban lineages (wild-caught *n* = 114, common garden *n* = 16). If selection is acting primarily on functional variation associated with nonsynonymous polymorphisms within coding regions, we would expect to observe a significant excess of nonsynonymous variants within our CT_MAX_-associated candidate SNP set when compared to the transcriptomic background. Alternatively, if selection is being driven primarily by genetic variation in *cis*-regulatory elements, we may expect a significant excess of noncoding polymorphisms.

**Fig. 8:**
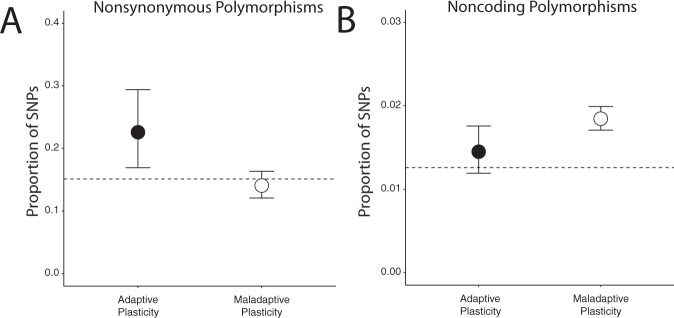
Evidence for different targets of selection on adaptive and maladaptive plasticity. **A** Single-nucleotide polymorphisms (SNPs)-associated adaptive plasticity display a significant enrichment of nonsynonymous polymorphisms, whereas those associated with maladaptive plasticity do not. **B** Single-nucleotide polymorphisms-associated maladaptive plasticity display a significant enrichment of noncoding polymorphisms), whereas those associated with adaptive plasticity do not. Dots and bars represent the proportion of SNPs among genes associated with adaptive (black) and maladaptive (white) plasticity, and 95% confidence intervals, respectively. Horizontal dotted lines represent expected proportions estimated from transcriptome-wide background (SNPs among genes unassociated with heat tolerance). All 130 animals for which we had sequence data were used in this analysis.

We found that genetic variation within genes putatively displaying adaptive plasticity contained a significant excess of nonsynonymous polymorphisms when compared to the background transcriptome (equality of proportions test: *χ*^2^ = 7.50; df = 1; *p* = 0.006, Fig. 8A). In contrast, genes displaying putatively maladaptive plasticity displayed no such enrichment (*χ*^2^ = 0.83; df = 1; *p* = 0.36, Fig. 8A). These results suggest that function-altering coding variation is the primary target of selection for adaptive plasticity in urban heat islands. The lack of enrichment of nonsynonymous variation within genes displaying maladaptive plasticity suggests, alternatively, that *cis-*regulatory variation may be the primary driver of selection against maladaptive plasticity.

To directly test this latter hypothesis, we identified SNPs within noncoding regions of the skeletal muscle transcriptome (putative *cis*-regulatory elements). We found that noncoding variants were overrepresented among genes displaying putatively maladaptive plasticity (*χ*^2^ = 96.646, df = 1, *p* << 0.001, Fig. 8B) when compared to the transcriptome-wide background. However, noncoding SNPs associated with adaptive plasticity display no significant enrichment over transcriptome-wide proportions (*χ*^2^ = 1.926, df = 1, *p* = 0.165, Fig. 8B). Taken together, these data suggest that adaptive and maladaptive plasticity may not only diverge at different rates across urban–forest boundaries, but that selection on adaptive and maladaptive plasticity may have occurred, at least in part, through different mechanisms in urban heat islands—with selection on beneficial coding mutations driving evolution favoring adaptive plasticity and selection against deleterious *cis*-regulatory variants promoting evolution to minimize and/or reverse maladaptive plasticity.

### Considerations and limitations of the methodological approach

Previous work has highlighted the complications of interpreting the relationship between ancestral plasticity and evolved divergence in transcriptomic data, as negative correlations emerge due to statistical artifact (i.e., regression towards the mean^79^). We have taken several measures in this study to account for the potential influence of this phenomenon. First, regression towards the mean is most likely to result when the most extreme cases in one comparison (i.e., differentially expressed genes between two groups) are reassessed in a subsequent comparison^79^. The identification of candidate genes in this study is not based on differential expression with regard to either ancestral plasticity nor evolved divergence, but on the strength of regulatory association with an environmentally relevant phenotype (CT_MAX_) with multiple lines of support as a target of selection in urban habitats^60^. In addition, randomization of the expression data reveal that the magnitude of correlation between ancestral plasticity and evolved divergence observed here is significantly greater than expected by chance^75,80^ (Fig. 6a inset). Most importantly, such statistical artifact arising from neutral variation in regulatory data is not expected to produce patterns of increased genetic divergence between lineages. The parallel signatures of increased genetic divergence among independently derived urban lineages further supports the role of natural selection in the evolution of the observed gene expression plasticity.

The major goal of this study is to understand the evolutionary mechanisms driving evolved divergence in gene expression associated with the observed difference in temperature-dependent performance between urban and forest anoles. Towards this goal, we test several independent but interrelated hypotheses to understand evolution of gene expression associated with differences in thermal tolerance between forest and urban populations in the wild. Our approach is dependent on isolating and focusing on gene expression patterns associated with thermal performance to assess patterns of evolved gene expression divergence that are relevant to the ecological context. These gene expression and sequence level analyses have provided multifaceted support for a significant evolutionary contribution to the observed difference in thermal performance observed in the wild.

However, our candidate gene approach likely only represents a partial picture of adaptive modification of a more complex, temperature-dependent phenotype (CT_MAX_). In order to gain a more complete understanding of the higher-order implications of lineage-specific divergence in expression among candidate genes and their underlying mechanisms, more extensive common garden study of whole organism thermal performance is needed^81^. Utilizing parallel changes across multiple urban–forest pairs, controlled breeding and experimental designs that isolate the impacts of developmental environment (developmental plasticity) and acute temperature change (phenotypic flexibility) on patterns of whole organism performance and its regulatory underpinnings would further contextualize the results of this study and provide greater insights regarding the importance of phenotypic plasticity as a means of rapid adaptation during the incipient stages of divergence. Nevertheless, the approaches presented herein demonstrate the utility of integrating data from field-based studies, common garden experiments, and multi-omics data, to reveal detailed mechanisms driving adaptive divergence between lineages. In this case, the results of our study provide fundamental insights into the relative roles of adaptive and maladaptive plasticity in driving the rapid evolution of complex regulatory systems in novel environments over anthropogenic timeframes.

Understanding the interactions between phenotypic plasticity and evolution remains an area of active debate within evolutionary biology^42,82^. This is largely due to a general lack of data regarding ancestral variation in environmentally induced plastic responses and limited information regarding their adaptive value or subsequent evolutionary impacts in the face of novel environmental challenges. Among studies that have focused on the evolution of plasticity, most have focused explicitly on adaptive plasticity. Although evolutionary change that reduces non-adaptive plasticity has been suggested as a common form of adaptation in the wild^83^, it has received relatively little attention^84^. Our findings support the role of phenotypic plasticity in adaptation to novel environments and highlight reduction/reversal of maladaptive plasticity as the predominant target of natural selection in urban heat islands. These findings are generally consistent with an emerging pattern: although adaptive plasticity facilitates survival and population persistence at early stages of colonization, genetic changes that minimize and/or reverse, rather than reinforce, phenotypic plasticity appear to be stronger and more frequent targets of selection^28,85–87^.

The common garden experimental design conducted in this study uses non-sibling individuals spread across temperature treatments. As a result, the plasticity reported herein represents plasticity of gene expression at the population level and does not enable us to quantify individual reaction norms or account for background genetic variation among individuals, which we acknowledge as a limitation of the study. However, population-level phenotypic differences observed in the wild are the focus of our investigation and despite high degree of gene flow between urban and forest habitats within each municipality, the populations are genetically differentiated. As such, the common garden experimental design does allow for interrogation of environmentally induced gene expression changes underpinning evolved divergence between habitat types.

The predominance of temperature-induced maladaptive plasticity observed in ancestral forest lineages suggests that thermal stress is a major selective pressure during the initial stages of urban colonization. The observed parallel patterns of genetic divergence are consistent with previous work on this system^60^ and suggest that behavioral thermoregulation is insufficient to shield these tropical urban populations from selection on temperature-dependent physiological performance^88,89^. The enrichment of gene ontology categories associated with heat shock protein transcription and DNA replication suggest that mediation of proteotoxicity due to the aggregation of misfolded proteins plays an important role in determining individual variation in ectothermic heat tolerance in urban heat islands. Together, this group of genes displays both broad signatures of polygenic selection across the island (consistent with thermal heterogeneity, and micro- and macroclimatic habitat specialization) and repeated divergence between urban and forest habitats. The maladaptive plasticity exhibited by forest lineages appears to be a primary target of natural selection in this latter context, resulting in a reduction and reversal^28^ of maladaptive heat-induced responses and a significant shift towards adaptive plasticity in urban lineages. Furthermore, selection on adaptive and maladaptive plasticity appear to occur via different genetic mechanisms—nonsynonymous and *cis*-regulatory variation, respectively.

The candidate gene approach that forms the foundation of this study uses the correlations between gene expression and CT_MAX_ to infer a group of regulatory drivers of whole organism performance. Although multiple lines of evidence presented here support these genes as direct targets of selection associated with thermal performance, further common garden study will be needed to directly quantify the evolution of thermal plasticity at the whole organism level. Indeed, the results of our study emphasize the necessity of simultaneous and integrative investigations of adaptive and non-adaptive plasticity across multiple levels of biological hierarchy to fully understand evolution and adaptation of complex traits in novel habitats. This integration of methods may be critical for predicting organismal response to anticipated anthropogenic habitat alteration and changes in future climate.

## Methods

Raw data collected for this study are the same as those in Campbell-Staton et al.^60^. All animal experiments were approved by the University of Massachusetts Boston under Institutional Animal Care and Use Committee (IACUC) protocol number 2012001. We summarize the data collection details here followed by the analytical methods unique to this study.

### Source populations

Gene expression analyses for this study were performed on two non-overlapping and unrelated groups of lizards: 114 wild individuals (“wild-caught” group) collected in 2016 and 16 captive, reared individuals (“common garden” group). Details of the experimental design and sample sizes are provided in Supplementary Data File 1.

The 114 wild-caught adult male *A. cristatellus* used in the study were captured using standard methods (hand capture and floss lasso) as encountered from paired urban and forest sites in four municipalities in Puerto Rico: Aguadilla, Arecibo, Mayagüez, and San Juan (Fig. 2A). Paired sites were within 10 km of each other. We placed lizards in cloth bags for transport to laboratory conditions where they were individually housed for the duration of the experiments. Lizards were allowed to acclimate to room temperature and humidity for 24 h prior to the initiation of thermal tolerance and subsequent acclimation experiments and transcriptome sequencing.

We established a captive common garden colony of *A. cristatellus* as part of a separate study^90^. In August 2013, we captured male–female pairs of adult *A. cristatellus* from the same urban site and a forest site sampled in 2016 in Mayagüez, Puerto Rico. We transported lizards to the animal care facility at the University of Massachusetts, Boston, where we paired individuals within their respective populations for breeding. We collected eggs from August 2013 to April 2014, placing them in hydrated vermiculate in an incubator (28 °C) until hatching. Hatchling lizards were immediately moved to individual cages in the animal care facility. All lizards were kept at a 13 h daylight schedule with a constant day/night temperature of 26.5 °C, daily misting, and vitamin dusted crickets provided every 3 days. All cages were rearranged regularly in the animal facility to minimize any effects of localized thermal variation. In 2016, 16 individuals were chosen from unique families for inclusion in the present study. Common garden lizards selected for the present study were F1 generation individuals 3–3.5 years of age, reared under identical conditions, and no full or half siblings were included in any of the experiments presented herein.

### Thermal tolerance and acclimation

Full methods for all thermal tolerance trials are presented in ref. ^60^, but we present relevant methods for this study here. We estimated CT_MAX_ by heating lizards at a rate of 1 °C min^−1^, starting at room temperature, while monitoring body temperature with a thermocouple inserted into the cloaca. After initiation of thermal stress responses (gaping, panting, and lethargy), we tested the righting response by placing the lizard onto its back. The temperature at which the lizard was no longer able to right itself within 30 s was recorded as its CT_MAX_, at which point the lizard was immediately cooled to room temperature in a water bath. CT_MAX_ estimates generally ranged between 35 °C and 43 °C. Thermal trials were conducted on all 130 lizards of this study (114 wild-caught, 16 common garden, see Supplementary Fig. 4 and Supplementary Data File 1).

Lizards were allowed a 24 h recovery period following CT_MAX_ trials. We then subjected wild-caught lizards to one of three thermal acclimation conditions for 2 h, approximating nocturnal forest (15 °C ± 1.82 °C), diurnal forest (25.4 °C ± 0.614 °C), or diurnal urban (32.09 °C ± 3.1 °C) conditions. Common garden animals were only subjected to the diurnal forest or diurnal urban acclimations. Following the acclimation period, lizards were anesthetized with 5% aerial isoflurane and killed via cervical dislocation. We immediately collected skeletal muscle from hind limbs and stored tissues in RNAlater at −80 °C. Thermal acclimation treatments were conducted for 130 lizards (114 wild-caught, 16 common garden, see Supplementary Fig. 4 and Supplementary Data File 1).

### Transcriptome sequencing and filtering

Total RNA was extracted from 130 tissue samples (114 wild-caught, 16 common garden) using Qiagen RNeasy Fibrous Tissue Kits. Messenger RNA libraries were prepared and sequenced for 100 base pair (bp) single-end reads on the Illumina Hiseq 4000, resulting in an average of ~22 M ± 2.6 reads. Trimmomatic^91^ was used to assess read quality in 4 bp sliding windows and sequences were trimmed when Phred33 quality scores fell below an average of 15 within a window. Sequences were then mapped to the *Anolis carolinensis* genome^92^ using Tophat2^93^.

### Statistical analyses

WGCNA^65^ was used to obtain a correlation-based de novo regulatory architecture for the skeletal muscle transcriptome using all samples for which transcriptome data were available (*n* = 130; 114 wild-caught, 16 common garden, Supplementary Fig. 1). Regulatory modules were identified as branches of the resulting cluster tree via the dynamic tree-cutting method and highly correlated modules (*R*^2^ = 0.75) were merged. The GS function in WGCNA was used to identify genes associated with individual variation in CT_MAX_, correcting for multiple testing within each identified regulatory module. An identical procedure was used to validate the candidate gene set, using only individuals from common garden (Supplementary Fig. 2) and wild-caught (Supplementary Fig. 3) animals, respectively.

Gene Ontology enrichment was performed us the gProfileR package in R^94^, using an unordered query analysis with a false discovery rate correction. The list of all genes represented within the skeletal muscle transcriptome was used as a customized background for analysis. All calculations of log-fold change in expression were obtained using the *edge*R software package in R^95^. Filtered RNAseq reads were mapped to the *A. carolinensis* reference genome with STAR v.2.5.b2^96^ using a single-pass approach with Ensembl v.90 annotations, retaining reads that were uniquely mapped with fewer than ten mismatches.

For sequence level analyses in this study, we used all individuals from Campbell-Staton et al.^60^ for which RNAseq data were available (*n* = 130; 114 wild-caught, 16 common garden). Variants were called with the GATK HaplotypeCaller and GenotypeGVCF v.3.5^97^ using the recommended RNAseq parameters (-stand_call_conf 20 -stand_emit_conf 20 —dontUseSoftClippedBases) and filtered for binary SNPs with quality ≥ 20, minor allele frequency ≥ 5%, and <20% missing data. Genetic divergence estimates were calculated using the analysis of molecular variance (AMOVA) method with a 5 Mb sigma parameter for smoothing within the Stacks software package (version 1.47)^98^. LD analyses were analyzed for 1 kb bins for each collection site using linear mixed-effects models. Pairwise LD was used as the response variable and gene set (a priori vs. background) was assigned as the predictor variable. Both SNP positions in each comparison were modeled separately as random effects. Differences in genetic divergence (*F*_ST_) were analyzed by Welch two-sample *t*-test. HW deviations were calculated with vcftools^99^ and differences in proportions were estimated by two-sample test for equality of proportions with continuity correction. Enrichment of nonsynonymous and noncoding SNPs among groups was assessed by two-sample equality of proportions test with continuity correct, using proportions obtained from the background data set as the null hypothesis. All analyses were conducted with the R statistical software package^100^.

Gene expression data were analyzed using the R package *edgeR*^95^. Raw read counts were first normalized by total library size and log-transformed using the *edgeR* functions *calcNormFactors* and *cpm*, respectively. The correlation between evolved divergence (average log-fold change in expression between forest vs. urban lizards at 32 °C) and ancestral plasticity (log-fold change between forest lizards acclimated to 25 °C vs. 32 °C) were examined with Spearman’s rank correlation coefficient (*ρ*) in *cor.test* in R. To account for potential statistical artifact in the regression, a null distribution of correlation coefficients was produced using a randomization and resampling prodecure: normalized gene expression data among the candidate gene set was randomized 1000 times using the *randomizeMatrix* function in the R package *picante*^101^, after which we recalculated average log-fold change in expression for ancestral plasticity, evolved divergence, and Spearman’s *ρ*. The upper and lower 95% limits of the null distribution were −0.48 and −0.59, respectively (Fig. 3A inset).

### Reporting summary

Further information on research design is available in the Nature Research Reporting Summary linked to this article.

## Supplementary information

Supplementary Information

Description of Additional Supplementary Files

Supplementary Data 1

Reporting Summary

## Data Availability

The sequence and metadata data used in this study are available in the NCBI database under accession code PRJNA592594. The data can be directly accessed at.  Source data are provided with this paper.

## Source data

Source Data

## Code availability

All statistical analyses have been conducted according to methods outlined herein, implemented in the associated referenced software packages. Code for reproduction of the figures herein are provided in the supplemental materials.

## References

[1] Grant, V. *Organismic Evolution* (Freeman, 1977).

[2] Falconer, D. *Introduction to Quantitative Genetics* (Longmans, 1981).

[3] Levin, D. in *Plant Evolutionary Biology* pp. 305–329 (Chapman and Hall, 1988).

[4] Ghalambor CK, McKay JK, Carroll SP, Reznick DN (2007). Adaptive versus non-adaptive phenotypic plasticity and the potential for contemporary adaptation in new environments. Funct. Ecol..

[5] Wright S (1931). Evolution in Mendelian populations. Genetics.

[6] Simpson G (1953). The Baldwin effect. Evolution.

[7] Williams, G. C. *Adaptation and Natural Selection* (Princeton Univ. Press, 1966).

[8] Kingsolver JG, Huey RB (1998). Evolutionary analyses of morphological and physiological plasticity in thermally variable environments. Am. Zool..

[9] Woods HA, Harrison JF (2002). Interpreting rejections of the beneficial acclimation hypothesis: When is physiological plasticity adaptive?. Evolution.

[10] Meyer A (1987). Phenotypic plasticity and heterochrony in *Cichlasoma managuense* (Pisces, Chichlidae) and their implications for speciation in cichlid fishes. Evolution.

[11] Losos JB (2000). Evolutionary implications of phenotypic plasticity in the hindlimb of the lizard *Anolis sagrei*. Evolution.

[12] Kappeler PM, Fichtel C (2015). Eco-evo-devo of the lemur syndrome: did adaptive behavioral plasticity get canalized in a large primate radiation?. Front. Zool..

[13] Nunney L, Cheung W (1997). The effect of temperature on body size and fecundity in female *Drosophila melanogaster*: evidence for adaptive plasticity. Evolution.

[14] Price TD, Qvarnström A, Irwin DE (2003). The role of phenotypic plasticity in driving genetic evolution. Proc. R. Soc. B Biol. Sci..

[15] Corl A (2018). The genetic basis of adaptation following plastic changes in coloration in a novel environment. Curr. Biol..

[16] Levis NA, Isdaner AJ, Pfennig DW (2018). Morphological novelty emerges from pre-existing phenotypic plasticity. Nat. Ecol. Evol..

[17] Whitehead A, Roach JL, Zhang S, Galvez F (2011). Genomic mechanisms of evolved physiological plasticity in killifish distributed along an environmental salinity gradient. Proc. Natl Acad. Sci. USA.

[18] Grant, P. R. & Grant, B. R. *Evolutionary Dynamics of a Natural Population* (Univ. Chicago Press, 1989).

[19] Huey, R. B. & Berrigan, D. in *Animals and Temperature: Phenotypic and Evolutionary Adaptation* pp. 205–238 (Cambridge Univ. Press, 1996).

[20] Blanckenhorn WU (2000). Temperature effects on egg size and their fitness consequences in the yellow dung fly *Scathophaga stercoraria*. Evol. Ecol..

[21] Woods HA, Harrison JF (2001). The beneficial acclimation hypothesis versus acclimation of specific traits: physiological change in water-stressed *Manduca sexta* caterpillars. Physiol. Biochem. Zool..

[22] Storz JF, Scott GR, Cheviron ZA (2010). Phenotypic plasticity and genetic adaptation to high-altitude hypoxia in vertebrates. J. Exp. Biol..

[23] Durmowicz AG, Hofmeister S, Kadyraliev TK, Aldashev AA, Stenmark KR (1993). Functional and structural adaptation of the yak pulmonary circulation to residence at high altitude. J. Appl. Physiol..

[24] Ge RL, Kubo K, Kobayashi T, Sekiguchi M, Honda T (1998). Blunted hypoxic pulmonary vasoconstrictive response in the rodent *Ochotona curzoniae* (pika) at high altitude. Am. J. Physiol. Hear. Circ. Physiol..

[25] Sakai A (2003). Cardiopulmonary hemodynamics of blue-sheep, *Pseudois nayaur*, as high-altitude adapted mammals. Jpn J. Physiol..

[26] Beall CM (2007). Two routes to functional adaptation: Tibetan and andean high-altitude natives. Proc. Natl Acad. Sci. USA.

[27] Velotta, J. P., Ivy, C. M., Wolf, C. J., Scott, G. R. & Cheviron, Z. A. Maladaptive phenotypic plasticity in cardiac muscle growth is suppressed in high-altitude deer mice. *Evolution* **72**, 2712–2727 (2018). 10.1111/evo.13626 30318588

[28] Ho WC, Zhang J (2018). Evolutionary adaptations to new environments generally reverse plastic phenotypic changes. Nat. Commun..

[29] Santangelo,J. S., Ruth Rivkin, L. & Johnson, M. T. J. The evolution of city life. *Proc. R. Soc. B Biol. Sci*. **285**, 10.1098/rspb.2018.1529 (2018). 10.1098/rspb.2018.1529 PMC6111153 30111603

[30] Thompson KA, Rieseberg LH, Schluter D (2018). Speciation and the city. Trends Ecol. Evol..

[31] Chown SL, Slabber S, McGeoch MA, Janion C, Leinaas HP (2007). Phenotypic plasticity mediates climate change responses among invasive and indigenous arthropods. Proc. R. Soc. B Biol. Sci..

[32] Charmantier A (2008). Adaptive phenotypic plasticity in response to climate change in a wild bird population. Science.

[33] Merilä J, Hendry AP (2014). Climate change, adaptation, and phenotypic plasticity: the problem and the evidence. Evol. Appl..

[34] Valladares F (2014). The effects of phenotypic plasticity and local adaptation on forecasts of species range shifts under climate change. Ecol. Lett..

[35] Nicotra AB (2010). Plant phenotypic plasticity in a changing climate. Trends Plant Sci..

[36] Oke T (1973). City size and the urban heat island. Atmos. Environ..

[37] Angilletta MJ (2007). Urban physiology: city ants possess high heat tolerance. PLoS ONE.

[38] Brans KI (2017). The heat is on: genetic adaptation to urbanization mediated by thermal tolerance and body size. Glob. Chang. Biol..

[39] Diamond SE, Chick L, Perez A, Strickler SA, Martin RA (2017). Rapid evolution of ant thermal tolerance across an urban-rural temperature cline. Biol. J. Linn. Soc..

[40] Hamblin AL, Youngsteadt E, Frank SD (2018). Wild bee abundance declines with urban warming, regardless of floral density. Urban Ecosyst..

[41] Diamond, S. E., Chick, L. D., Perez, A., Strickler, S. A. & Martin, R. A. Evolution of thermal tolerance and its fitness consequences: parallel and non-parallel responses to urban heat islands across three cities. *Proc. R. Soc. B Biol. Sci*. **285**, 10.1098/rspb.2018.0036 (2018). 10.1098/rspb.2018.0036 PMC6053939 30051828

[42] Gibert P, Debat V, Ghalambor CK (2019). Phenotypic plasticity, global change, and the speed of adaptive evolution. Curr. Opin. Insect Sci..

[43] Chick LD, Strickler SA, Perez A, Martin RA, Diamond SE (2019). Urban heat islands advance the timing of reproduction in a social insect. J. Therm. Biol..

[44] Pipoly I, Bókony V, Seress G, Szabó K, Liker A (2013). Effects of extreme weather on reproductive success in a temperate-breeding songbird. PLoS ONE.

[45] Tiatragul S, Kurniawan A, Kolbe JJ, Warner DA (2017). Embryos of non-native anoles are robust to urban thermal environments. J. Therm. Biol..

[46] Kaiser A, Merckx T, Van Dyck H (2016). The urban heat island and its spatial scale dependent impact on survival and development in butterflies of different thermal sensitivity. Ecol. Evol..

[47] Hall JM, Warner DA (2018). Thermal spikes from the urban heat island increase mortality and alter physiology of lizard embryos. J. Exp. Biol..

[48] Johnson JC, Urcuyo J, Moen C, Stevens DR (2019). Urban heat island conditions experienced by the Western black widow spider (*Latrodectus hesperus*): extreme heat slows development but results in behavioral accommodations. PLoS ONE.

[49] Battles AC, Kolbe JJ (2019). Miami heat: urban heat islands influence the thermal suitability of habitats for ectotherms. Glob. Chang. Biol..

[50] Hamblin, A. L., Youngsteadt, E., López-Uribe, M. M. & Frank, S. D. Physiological thermal limits predict differential responses of bees to urban heat-island effects. *Biol. Lett*. **13**, 10.1098/rsbl.2017.0125 (2017). 10.1098/rsbl.2017.0125 PMC5493736 28637837

[51] Kingsolver JG, Diamond SE, Buckley LB (2013). Heat stress and the fitness consequences of climate change for terrestrial ectotherms. Funct. Ecol..

[52] Huey RB, Hertz PE, Sinervo B (2003). Behavioral drive versus behavioral inertia in evolution: a null model approach. Am. Nat..

[53] Bogert CM (1949). Thermoregulation in reptiles, a factor in evolution. Evolution.

[54] Wingfield JC, Sapolsky RM (2003). Reproduction and resistance to stress: when and how. J. Neuroendocrinol..

[55] Angilletta MJ (2009). Looking for answers to questions about heat stress: researchers are getting warmer. Funct. Ecol..

[56] James CD, Whitford WG, James CD, Whitford WG (1994). An experimental study of phenotypic plasticity in the clutch size of a lizard. Oikos.

[57] Sorci G, Clobert J, Belichon S (1996). Phenotypic plasticity of growth and survival in the common lizard *Lacerta vivipara*. J. Anim. Ecol..

[58] Jordan MA, Snell HL (2002). Life history trade-offs and phenotypic plasticity in the reproduction of *Galápagos lava* lizards (*Microlophus delanonis*). Oecologia.

[59] Gilbert AL, Miles DB (2019). Antagonistic responses of exposure to sublethal temperatures: adaptive phenotypic plasticity coincides with a reduction in organismal performance. Am. Nat..

[60] Campbell-Staton SC (2020). Parallel selection on thermal physiology facilitates repeated adaptation of city lizards to urban heat islands. Nat. Ecol. Evol..

[61] Herrel A, Vanhooydonck B, Porck J, Irschick D (2008). Anatomical basis of differences in locomotor behavior in *Anolis* lizards: a comparison between two ecomorphs. Bull. Mus. Comp. Zool..

[62] Anderson CV, Roberts TJ (2020). The need for speed: functional specializations of locomotor and feeding muscles in *Anolis* lizards. J. Exp. Biol..

[63] Cowles R, Bogert C (1944). A preliminary study of the thermal requirements of desert reptiles. Bull. Am. Mus. Nat. Hist..

[64] Lutterschmidt WI, Hutchison VH (1997). The critical thermal maximum: data to support the onset of spasms as the definitive end point. Can. J. Zool..

[65] Langfelder P, Horvath S (2008). WGCNA: an R package for weighted correlation network analysis. BMC Bioinformatics.

[66] Cardiello JF, Goodrich JA, Kugel JF (2018). Heat shock causes a reversible increase in RNA polymerase II occupancy downstream of mRNA genes, consistent with a global loss in transcriptional termination. Mol. Cell. Biol..

[67] Sandaltzopoulos R, Becker PB (1998). Heat shock factor increases the reinitiation rate from potentiated chromatin templates. Mol. Cell. Biol..

[68] Velichko AK, Petrova NV, Kantidze OL, Razin SV (2012). Dual effect of heat shock on DNA replication and genome integrity. Mol. Biol. Cell..

[69] Barreiro LB, Laval G, Quach H, Patin E, Quintana-Murci L (2008). Natural selection has driven population differentiation in modern humans. Nat. Genet..

[70] Huey, R. B. & Webster, T. P. Thermal biology of *Anolis* lizards in a complex fauna: the *Christatellus* group on Puerto Rico. *Ecology* **57**, 985–994 http://www.jstor.org/stable/1941063 (1976).

[71] Gorman GC, Hillman S (1977). Physiological basis for climatic niche partitioning in two species of Puerto Rican *Anolis* (*Reptilia*, *Lacertilia*, *Iguanidae*). J. Herp.

[72] Gunderson, A. R., Mahler, D. L. & Leal, M. Thermal niche evolution across replicated *Anolis* lizard adaptive radiations. *Proc. R. Soc. B Biol. Sci*. **285**, 10.1098/rspb.2017.2241 (2018). 10.1098/rspb.2017.2241 PMC5936720 29669895

[73] McKechnie AE, Wolf BO (2010). Climate change increases the likelihood of catastrophic avian mortality events during extreme heat waves. Biol. Lett..

[74] Huey RB, Losos JB, Moritz C (2010). Are lizards toast?. Science.

[75] Ghalambor CK (2015). Non-adaptive plasticity potentiates rapid adaptive evolution of gene expression in nature. Nature.

[76] Van Gestel J, Weissing FJ (2018). Is plasticity caused by single genes?. Nature.

[77] Turchin MC (2012). Evidence of widespread selection on standing variation in Europe at height-associated SNPs. Nat. Genet..

[78] Guo J (2018). Global genetic differentiation of complex traits shaped by natural selection in humans. Nat. Commun..

[79] Mallard F, Jakšic´ AM, Schlötterer C (2015). Contesting the evidence for non-adaptive plasticity. Nature.

[80] Ghalambor CK (2015). Reply to Ghalambor et al. Nature.

[81] Perrier, C., Caizergues, A. & Charmantier, A. in *Urban Evolutionary Biology* (eds. Szulkin, M., Munshi-South, J. & Charmantier, A.) pp. 74–90 (Oxford Univ. Press, 2020).

[82] Lambert MR, Brans KI, Des Roches S, Donihue CM, Diamond SE (2021). Adaptive evolution in cities: progress and misconceptions. Trends Ecol. Evol..

[83] Grether, G. F. Environmental change, phenotypic plasticity, and genetic compensation. *Am. Nat*. **166**, 10.1086/432023 (2005). 10.1086/432023 16224697

[84] Velotta JP, Cheviron ZA (2018). Remodeling ancestral phenotypic plasticity in local adaptation: a new framework to explore the role of genetic compensation in the evolution of homeostasis. Integr. Comp. Biol..

[85] Fischer EK, Ghalambor CK, Hoke KL (2016). Can a network approach resolve how adaptive vs nonadaptive plasticity impacts evolutionary trajectories?. Integr. Comp. Biol..

[86] Huang Y, Agrawal AF (2016). Experimental evolution of gene expression and plasticity in alternative selective regimes. PLoS Genet..

[87] Leonard AM, Lancaster LT (2020). Maladaptive plasticity facilitates evolution of thermal tolerance during an experimental range shift. BMC Evol. Biol..

[88] Kearney, M., Shine, R. & Porter, W. P. The potential for behavioral thermoregulation to buffer “cold-blooded” animals against climate warming. *Proc. Natl Acad. Sci. USA* **106**, 3835–3840 (2009). 10.1073/pnas.0808913106 PMC2656166 19234117

[89] Huey RB, Tewksbury JJ (2009). Can behavior douse the fire of climate warming?. Proc. Natl Acad. Sci. USA.

[90] Winchell KM, Reynolds RG, Prado-irwin SR, Puente-rol AR, Revell LJ (2016). Phenotypic shifts in urban areas in the tropical lizard *Anolis cristatellus*. Evolution.

[91] Bolger AM, Lohse M, Usadel B (2014). Trimmomatic: a flexible trimmer for Illumina sequence data. Bioinformatics.

[92] Alföldi J (2011). The genome of the green anole lizard and a comparative analysis with birds and mammals. Nature.

[93] Kim D (2013). TopHat2: accurate alignment of transcriptomes in the presence of insertions, deletions and gene fusions. Genome Biol..

[94] Reimand J (2007). g:Profiler—web-based toolset for functional profiling of gene lists from large-scale experiments. Nucleic Acids Res..

[95] Robinson MD, Mccarthy DJ, Smyth GK (2010). edgeR: a Bioconductor package for differential expression analysis of digital gene expression data. Bioinformatics.

[96] Dobin A (2013). STAR: ultrafast universal RNA-seq aligner. Bioinformatics.

[97] McKenna, D. M. et al. The genome analysis toolkit: a MapReduce framework for analyzing next-generation DNA sequencing data. *Genome Res*. **20**, 1297–1303 (2010). 10.1101/gr.107524.110 PMC2928508 20644199

[98] Catchen J, Hohenlohe PA, Bassham S, Amores A, Cresko WA (2013). Stacks: an analysis tool set for population genomics. Mol. Ecol..

[99] Danecek P (2011). The variant call format and VCFtools. Bioinformatics.

[100] R Foundation for Statistical Computing. *R: A Language and Environment for Statistical Computing*, https://www.r-project.org (2017).

[101] Kembel SW (2010). Picante: R tools for integrating phylogenies and ecology. Bioinformatics.

