# Supplementary material for: Selection on adaptive and maladaptive gene expression plasticity during thermal adaptation to urban heat islands
Source: Nat Commun. 2021 Oct 26;12:6195. doi: 10.1038/s41467-021-26334-4 (PMC8548502; doi:10.1038/s41467-021-26334-4)
Supplement: Supplementary file 1 — Supplementary Information [file 41467_2021_26334_MOESM1_ESM.pdf]

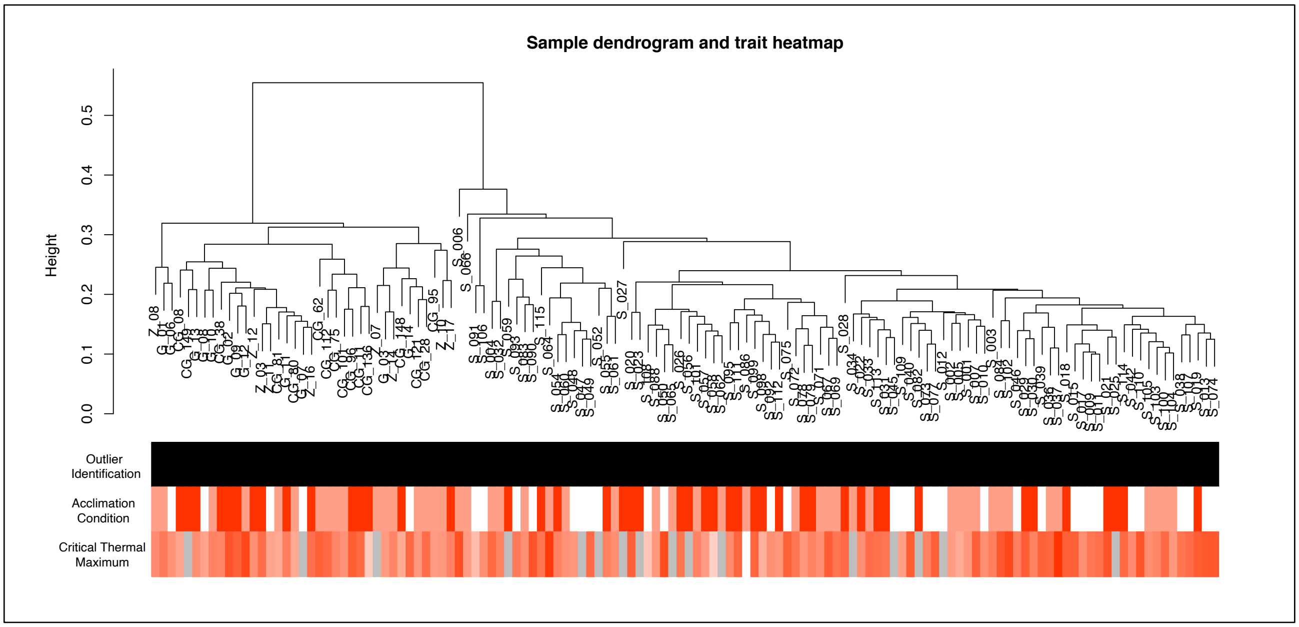

Supplemental Figure 1: WGCNA Sample Dendrogram. Dendrogram depicts all samples included in WGCNA analyses to generate co-regulatory modules and identify CT_{MAX}-associated genes. The top row ("Outlier Identification") indicates that no samples are outliers in their overall expression profiles and all samples were retained for analyses. The middle row indicates the acclimation condition of each individual: 15°C (white), 25°C (pink) or 32°C (red). The bottom row indicates relative CT_{MAX} measured for a given individual, with warmer colors indicating higher heat tolerance. Grey bars indicate individuals for which CT_{MAX} data were unavailable.

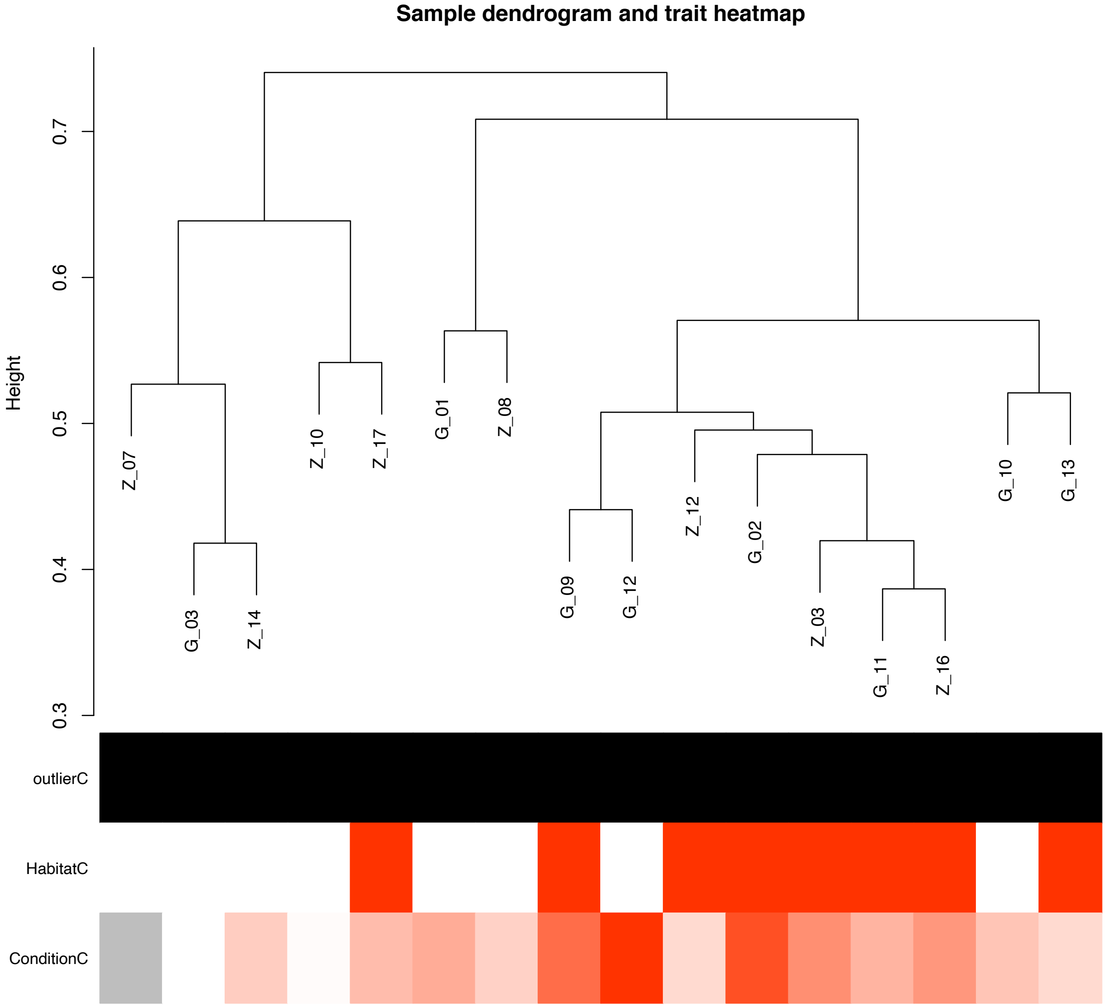

Supplemental Figure 2: Common Garden WGCNA Sample Dendrogram. Dendrogram depicts all common garden samples included in WGCNA analyses to generate co-regulatory modules and identify CT_{MAX}-associated genes. The top row ("Outlier Identification") indicates that no samples are outliers in their overall expression profiles and all samples were retained for analyses. The middle row indicates the acclimation condition of each individual. Common garden animals were only subjected to two acclimation conditions: 25°C (white), or 32°C (red). The bottom row indicates relative CT_{MAX} measured for a given individual, with warmer colors indicating higher heat tolerance. Grey bars indicate individuals for which CT_{MAX} data were unavailable.

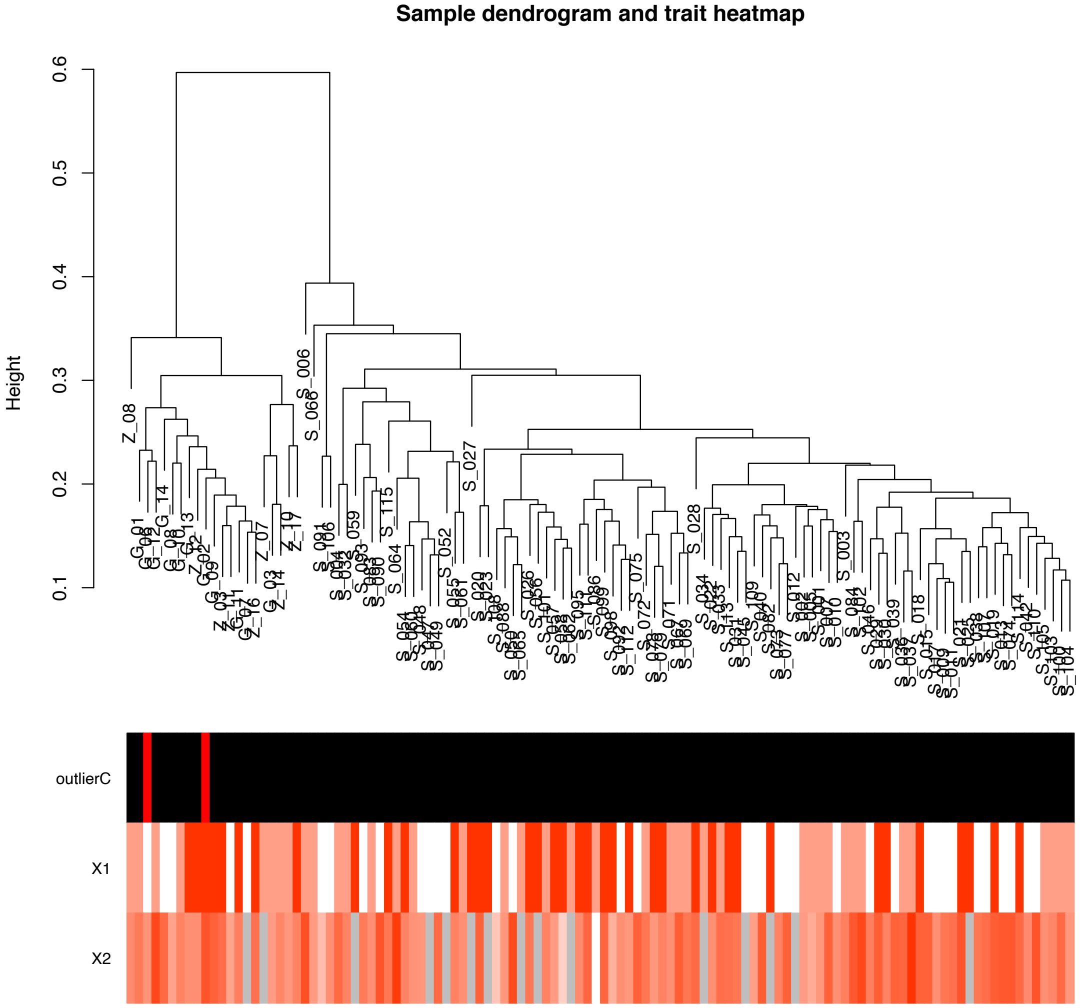

Supplemental Figure 3: Wild-Caught WGCNA Sample Dendrogram. Dendrogram depicts all wild-caught samples included in WGCNA analyses to generate co-regulatory modules and identify CT_{MAX}-associated genes. The top row indicates that two samples are outliers in their overall expression profiles and these samples were removed before downstream analyses. The middle row indicates the acclimation condition of each individual: 15°C (white), 25°C (pink) or 32°C (red). The bottom row indicates relative CT_{MAX} measured for a given individual, with warmer colors indicating higher heat tolerance. Grey bars indicate individuals for which CT_{MAX} data were unavailable.

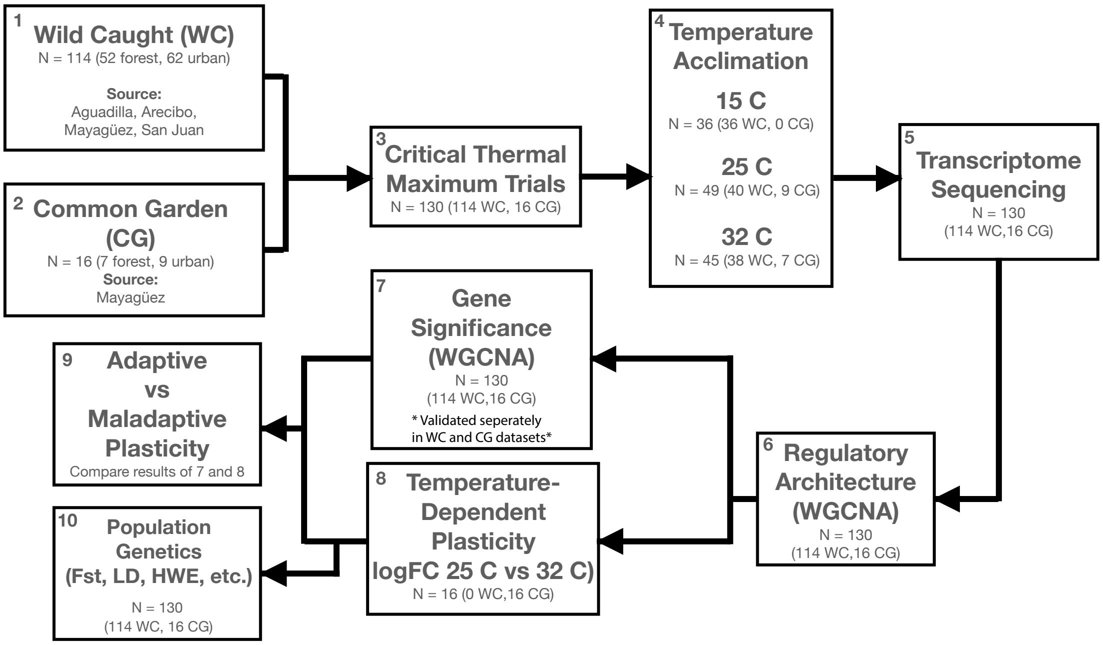

Supplemental Figure 4: Data workflow. Workflow of analyses performed in this study with samples sizes and origins provided at each step.

**Municipality**	**Est.**	**Urban Establishment (source)**	**Dist. (km)**	**Exp.**	**Habitat**	**Lat., Long.**
Aguadilla	1775	1936 (Est. of Ramey AFB)	3.4	Wild	Urban	18.50330, -67.13477
					Forest	18.48686, -67.16164
Arecibo	1616	1980 (Est. of Interamerican University Campus)	7.6	Wild	Urban	18.47525, -66.75880
					Forest	18.41065, -66.72647
Mayagüez	1760	<1958 (earliest aerial imagery available)	4.4	Wild CG	Urban	18.21439, -67.14731
					Forest	18.23357, -67.11017
San Juan	1509	<1962 (earliest aerial imagery available)	7.4	Wild	Urban	18.45318, -66.06302
					Forest	18.38648, -66.05330

Supplemental Table 1: Paired urban and forest ample sites in Puerto Rico from four municipalities, with founding date of the municipality (Est.), estimated date urban habitat was established with source of estimate, distance between urban and forest pairs within each municipality, experiment populations were included in (Exp.), habitat type, and latitude and longitude of each site.

Supplemental Table 2: Gene Ontology Enrichment Analysis of CT_{MAX}-associated genes

significant	p.value	term.size	query.size	overlap.size	precision	recall	term.id	domain	subgraph.number	term.name	relative.depth	intersection
TRUE	0.0359	30	233	7	0.03	0.233	GO:0044089	BP	7	positive regulation of cellular component biogenesis	1	SACAG00000006812,ENSACAG00000007383,ENSAC AG00000014583,ENSACAG00000015214,ENSACAG [illegible]
TRUE	0.00381	245	233	28	0.12	0.114	GO:0044265	BP	4	cellular macromolecule catabolic process	1	SACAG00000001021,ENSACAG00000002289,ENSAC AG00000003160,ENSACAG00000003508,ENSACAG 00000003615,ENSACAG00000004352,ENSACAG000 00004409,ENSACAG00000004732,ENSACAG000000 06670,ENSACAG00000006742,ENSACAG000000068 12,ENSACAG00000007067,ENSACAG00000008845,
TRUE	0.00035	6	233	5	0.021	0.833	GO:0045898	BP	3	regulation of RNA polymerase II transcriptional preinitiation complex assembly	1	SACAG00000006812,ENSACAG00000014583,ENSAC AG00000029385
TRUE	0.0254	46	233	9	0.039	0.196	GO:0007059	BP	1	chromosome segregation	1	SACAG00000001904,ENSACAG00000003477,ENSAC AG00000006621,ENSACAG00000008766,ENSACAG
TRUE	7.65E-09	19	233	12	0.052	0.632	GO:0005838	CC	5	proteasome regulatory particle	1	SACAG00000002289,ENSACAG00000003160,ENSAC AG00000004732,ENSACAG00000006670,ENSACAG 00000006812,ENSACAG00000013643,ENSACAG000
TRUE	0.00035	6	233	5	0.021	0.833	GO:0036402	MF	6	proteasome-activating ATPase activity	1	SACAG00000006812,ENSACAG00000014583,ENSAC AG00000029385
TRUE	0.00381	9	233	5	0.021	0.556	GO:0017025	MF	2	TBP-class protein binding	1	SACAG00000006812,ENSACAG00000014583,ENSAC AG00000029385

Municipality	Habitat	Background Set Proportion	Candidate Set Proportion	X^2	df	p-value
Aguadilla	Forest	0.042	0.083	77.72	1	< 2.2e-16
Aguadilla	Urban	0.054	0.083	28.478	1	9.475e-08
Arecibo	Forest	0.057	0.086	29.763	1	4.883e-08
Arecibo	Urban	0.058	0.085	23.702	1	1.125e-06
Mayagüez	Forest	0.052	0.076	20.795	1	5.113e-06
Mayagüez	Urban	0.052	0.084	37.71	1	8.208e-10
San Juan	Forest	0.042	0.066	25.596	1	4.208e-07
San Juan	Urban	0.058	0.089	33.383	1	7.57e-09

Supplemental Table 3: Comparisons of the proportion of SNPs within candidate and background gene sets displaying significant deviations ($p < 0.05$) from Hardy-Weinberg Equilibrium in each population studied. Analysis was performed on all 150 animals for which we had sequence data (wild-caught = 114, common garden = 16).

Glossary of Key Terms

Adaptive plasticity: Any environmentally induced trait/trait value that has a positive effect on fitness

Ancestral plasticity: Phenotypic plasticity present in an ancestral population

Maladaptive plasticity: A subset of non-adaptive plasticity in which an environmentally induced trait/trait value has a negative effect on fitness

Non-adaptive plasticity: A general term referring to any phenotypic plasticity that has no effect or a negative effect on fitness

Phenotypic plasticity: The phenomenon by which a single genotype produces two or more phenotypes in response to an environmental stimulus
