# Supplementary material for: Selection on adaptive and maladaptive gene expression plasticity during thermal adaptation to urban heat islands
Source: Nat Commun. 2021 Oct 26;12:6195. doi: 10.1038/s41467-021-26334-4 (PMC8548502; doi:10.1038/s41467-021-26334-4)
Supplement: Supplementary file 2 — Description of Additional Supplementary Files [file 41467_2021_26334_MOESM2_ESM.pdf]

Description of Additional Supplementary Files

File name: Supplementary Data 1

Description: All samples used in the Main Text and supplemental analyses. Columns represent all analyses performed in the manuscript. All samples used in each analysis are indicated by 'Yes' in their respective columns. Samples not used in a given analysis are indicated by 'No'.
